# Unraveling the physicochemical attributes of three cricket (*Gryllus bimaculatus*)-enriched biscuit products and implications on consumers’ preference and willingness to pay

**DOI:** 10.1016/j.lwt.2023.115171

**Published:** 2023-08-01

**Authors:** Divina Arama, John Kinyuru, Jeremiah Ng'ang'a, Beatrice Kiage-Mokua, Brian O. Ochieng, Chrysantus Mbi Tanga

**Affiliations:** a International Centre of Insect Physiology and Ecology (*icipe*), P.O. Box 30772-00100, Nairobi, Kenya; b Department of Human Nutrition Sciences, Jomo Kenyatta University of Agriculture and Technology, P.O Box 62000-00200, City Square Nairobi, Kenya

**Keywords:** Cricket meal, Food supplementation, Nutritional quality, Sensory evaluation, Caregivers, Children, Household food security

## Abstract

Disgust and neophobia elicited by whole insect products, has necessitated the need to mask insect-based food products. The physico-chemical parameters, sensory acceptance, and willingness to pay (WTP) for wheat biscuits supplemented with cricket powder was evaluated. The biscuits’ color intensity correlated with the cricket inclusion levels. Spread ration of cricket-enriched-biscuits increased (1.0–1.2-folds), while the hardness and fracturability decreased (1.0–1.3-folds and 1.0–1.2 folds, respectively) compared to the control biscuit. Cricket-biscuits exhibited 1.2–1.7, 1.1–3.7, 1.2–3.0 and 1.1–1.2-folds higher (p < 0.05) protein, ash, fiber, and fat, respectively. Ca, Fe, and Zn were 1.1–3.7, 1.1–1.2 and 1.4–4.0-folds higher, respectively, for cricket-based biscuits. Monounsaturated and polyunsaturated fatty acids proportionally increased with increasing cricket flour. On a likert scale, 71.4%, 71.9%, 38.4% and 57.5% of the caregivers and 38.6%, 58.3%, 40.0% and 34.0% for children (3–5 years) strongly preferred the color, texture, taste and aroma, respectively, of the cricket-based biscuits. Forty-seven (47%) of the caretakers were WTP a premium of 37 Kenyan shillings (0.34 USD) for cricket-based biscuits. Our findings demonstrated that integration of cricket flour into existing market-driven consumer familiar food products significantly increased acceptability and WTP, thus promising potential to contribute to improved food and nutritional security.

## Highlights

• Childhood malnutrition is an important public health problem.
• Protein and fat in cricket-enriched biscuits exceeded the daily recommended amount.
• Cricket-enriched biscuits can tackle protein-energy malnutrition, Fe, and Zn deficiencies.
• Caregivers expressed increased acceptability and WTP for cricket-enriched biscuits.
• More studies are needed to examine their effect on nutritional status in children.

## 1 Introduction

Food insecurity is a global challenge that has been exacerbated by climate change and rapid population growth (Wendin & Nyberg, 2021). Around 820 million people worldwide are food insecure (Biró et al., 2020), and over 256 million of these are in Africa (Hlongwane, Slotow, & Munyai, 2020). According to the Food and Agriculture Organization of the United Nations, around 28 million people are malnourished and on the verge of starvation in East Africa alone (FAO, 2021). The most vulnerable are children and breastfeeding mothers in low and middle-income countries (De & Chattopadhyay, 2019). Generally, fast growth and development depend on good nutrition in early childhood, which can only be kept up with the right amount and quality of food. Thus, the current food insecurity situation calls for urgent and sustainable strategies to fulfill the unmet food shortage, which is rampant in many developing countries (Akande, Jolayemi, Adelugba, & Akande, 2020).

Entomophagy (the consumption of insects) has been practiced in many civilizations around the world and is re-emerging as a source of high-quality, cost-effective, and sustainable alternative food sources to meat (Sogari, Bogueva, & Marinova, 2019; About 2000 insect species are consumed worldwide (Patel, 2019). Edible insects are widely available in most countries worldwide, have high reproduction rates (Kipkoech, Kinyuru, Imathiu, Meyer-Rochow, & Roos, 2021), high growth rates (Dobermann, Swift, & Field, 2017), high feed conversion rates (van Huis & Oonincx, 2017), low environmental footprint (van Huis, Rumpold, van der Fels-Klerx, & Tomberlin, 2021), and are significant sources of macro and micronutrients (Cheseto, Baleba, Tanga, Kelemu, & Torto, 2020). Their protein levels have been established to supersede or compare favorably with conventional proteins sources especially those of animal origin (Anankware et al., 2021).

Crickets are among the most consumed insects globally (Magara et al., 2021), due to their high nutritional quality (Tao & Li, 2018). Crickets contain all the nine necessary amino acids in correct proportions (Homann, Ayieko, Konyole, & Roos, 2017), and high levels of fat, calcium, potassium, zinc, magnesium, copper, and folate (Smarzyński et al., 2021). Crickets are also high in fiber, which is rare among animal proteins, with a content of about 13% (Magara et al., 2021). Improved digestive health has been associated with the consumption of chitin, an insoluble fiber found in crickets. Chitin acts as a prebiotic, encouraging the growth of beneficial microbes such as *Bifidobacterium animalis* in the digestive tract (Skotnicka, Karwowska, Kłobukowski, Borkowska, & Pieszko, 2021). In addition, consumption of crickets has been found to reduce inflammatory gastrointestinal problems and the risk of heart diseases. Moreover, chitin's ability to stimulate and control the immune system is well-documented. The lungs and gut are the primary sites of chitin sensation which subsequently triggers innate immune response including eosinophils and macrophages, and an adaptive immunological response involving T helper cells (Elieh Ali Komi, Sharma, & Dela Cruz, 2018).

The two-spotted cricket or field cricket (*Gryllus bimaculatus* De Geer) is one of the most widespread species worldwide and has been shown to be a viable source of food and feed (Van Huis, 2020). This species is favored primarily because it matures quickly, grows to a larger body size, and is more marketable (Van Huis, 2020). According to research by Murugu et al. (2021), *G. bimaculatus* has a crude protein level of 58% that is comparable to that of animal protein sources. Additionally, *G. bimaculatus* contains 6.90% crude fiber, 26.90% fat, and 78.90% of all digestible nutrients (Phesatcha, Phesatcha, Viennaxay, Matra, & Totakul, 2022). Additionally, higher levels of iron, zinc, and potassium in *G. bimaculatus* than in plant and animal sources has been reported (Magara et al., 2021). Cricket may be a desirable protein supplement, due to its high nutrient quality. According to a previous investigation by Seong and Kim (2021) when the cricket was added to brown rice, protein and total amino acid content increased significantly. Elsewhere, extracts from *G. bimaculatus* have been reported to effectively counteract steatosis and apoptosis associated with alcoholism as well as mitigating intestinal permeability to microbial toxins and suppressing cellular inflammations (Hwang et al., 2019). This suggests that *G. bimaculatus* is a potential source of therapeutic elements critical to advancing human medicine. The black cricket has been primarily farmed and utilized as human food (Murugu et al., 2021), despite the fact that very little information has been published regarding the possibility of its usage as a protein substitute in human diets.

Despite the numerous health benefits associated with crickets and cricket-based foods, the consumption of crickets is still low in most African countries, including Kenya (Magara et al., 2021). This has been largely attributed to psychological barriers to insect consumption such as disgust and neophobia, both of which have caused low acceptability (Verneau, La Barbera, Amato, Riverso, & Grunert, 2020). A person's tendency to reject food is strongly influenced by their level of disgust, which is engrained in their culture because food is defined by culture (Orsi, Voege, & Stranieri, 2019). Anxiety or even a fear of strange foods, such as insects that are regarded as dangerous, is linked to meal acceptability (Nyberg, Olsson, & Wendin, 2021). To increase the acceptability and consumption of insects and insect-based foods, researchers have recommended their integration into existing food products, especially bakery products, masking the physical characteristics of such a food source from the eyes of the consumers (Tan & House, 2018). Biscuits are a popular food product made with wheat flour, sugar, and fat as the key ingredients. Wheat flour is the main ingredient in biscuits, and starch is the main component, with fat and sugar also playing important roles (Schuster, Huen, & Scherf, 2023). Unlike other cereal flours, wheat flour makes viscoelastic dough when coupled with water due to the presence of gluten proteins, which are compatible with water and interact and swell to produce viscoelasticity (Schuster et al., 2023). Due to the fact that wheat is the primary ingredient, biscuits have an unbalance nutritional profiles (it is energy packed but not nutrient-dense) (Gómez, Gutkoski, & Bravo-Núñez, 2020), hence the inclusion of cricket in the biscuit dough mix could possibly enhance the nutritional value of this product. Moreover, the convenience of its formulation mix permits the incorporation of various ingredients, hence, can be utilized to convey ingredients with superior nutritional profiles. The potential use of cricket flour in wheat products has been investigated previously to create a variety of insect-based food products that are varied, nutritious, intuitive, and generally accepted. For example, cricket flour has been used to make products like gluten-free bread (Kowalczewski et al., 2021), oat biscuits (Biró et al., 2020), sponge cake (Vlahova-Vangelova, Balev, Kolev, Dinkova, & Stankov, 2022), and buns (Pambo, Okello, Mbeche, Kinyuru, & Alemu, 2018).

With the rife in food insecurity in developing countries, there is urgent need to diversify protein sources used to enrich common consumer friendly ready-to-eat snacks that are highly marketable. The present study provides three main contributions to consumer preferences research. First, previous studies have largely focused on willingness to eat, as well as product liking (Alemu, Olsen, Vedel, Pambo, & Owino, 2015; de-Magistris, Pascucci, & Mitsopoulos, 2015), rather than on the non-hypothetical willingness to pay (WTP) of caretakers for cricket-enriched food products. Second, we assess the benefits of integrating cricket meal in enhancing the nutrient quality of biscuits. Third, we contribute to consumer research on novel functional foods by investigating the role of cricket-enriched biscuits in influencing caregivers’ acceptance and WTP.

## 2 Material and methods

### 2.1 Cricket acquisition and biscuits formulation

All the ingredients used (all-purpose baking wheat flour, whole milk, margarine, table salt, sugar, and baking powder) were purchased from the local market. Six kilograms of crickets (*G. bimaculatus*), fed and bred on green leafy vegetables and chicken feed, were obtained from the Jomo Kenyatta University of Agriculture and Technology Insect Farm, Nairobi, Kenya and temporarily frozen-stored at −30 °C. The crickets were thawed, oven-dried at 180 °C for 20 min, ground in an electric grinder (Kenwood, Havant, UK) and sieved through 200-micron-sized mesh to attain consistent particle size. The cricket flour obtained was used to substitute wheat flour at varying proportions: 5% (CBB5), 10% (CBB10), and 20% (CBB20) with 0% (w/w) serving as control (no insect flour included). The basic nutritional characteristics of *G. bimaculatus* meal and wheat flour used in the biscuit's formulation are presented in Table 1 as adopted from Murugu et al. (2021) and Ochieng et al. (2023), respectively. Biscuits were prepared according to an existing recipe by Dewi, Vidiarti, Fitranti, Kurniawati, and Anjani (2020) with various modifications. Briefly, margarine (20 g) was mixed with dry ingredients: salt (0.45 g), sugar (25 g) and wheat-cricket flours blend or wheat flour for control (100 g) in a bowl of a kitchen aid mixer (HOBART, USA) at medium speed for 5 min. Whole milk (15 mL) was then added and mixed for another 1 min at low speed to obtain a smooth and elastic dough. The mixture was then hand-kneaded into consistent dough for 3 min, smoothed out thinly using a rolling pin to a thickness of approximately 50 mm and cut into round shapes of 60 mm diameter with a circular mould. The pieces were placed on an oiled baking pan and baked in a pre-heated oven (BISTROT 665; BestFor®, Ferrara, Italy) at 180 °C for 30 min baking of the cookies was repeated three times for each substitution level resulting into twelve sets of baked products. The baked biscuits were cooled, packaged in air tight polythene bags and kept at room temperature.

**Table 1:** Basic characteristics (proximate components) (% dry matter basis) of *Gryllus bimaculatus* and wheat flours utilized in biscuit preparation.

Flour type	Crude protein content	Crude fat content	Crude ash content	Crude fibre content	Carbohydrate
^§^*Gryllus bimaculatus*	58.2	46.0	5.4	8.4	N.D.
^⸸^Wheat flour	12.6	1.7	2.5	0.6	70.4

Source: (Murugu et al., 2021) ^§^; (Ochieng et al., 2023)^⸸^.

### 2.2 Assessment of the physical properties

Within 24 h of preparation, the weight, diameter, thickness, spread ratio, textural quality, and color of the different biscuits products were determined following standard methods described by Association of Official Agricultural Chemists (AOAC, 2010). Biscuits diameter and thickness were measured using a digital caliper (Z22855F, Bedfordshire, UK) whereas the weights were obtained using a digital weighing scale (CGOLDENWALL, USA). The spread ratio was calculated by dividing the diameter of the biscuits with their respective thicknesses. Biscuits color was determined using a Hunter's Lab color analyzer (Hunter Lab scan XE, VA, USA) by applying L*, a*, and b* color dimensions in evaluating the brightness parameters (L*) ranging from 0 (black) to 100 (white) and green-red (a*) and blue-yellow (b*) chromaticity. The overall color difference (ΔE) was computed using the formula below:

Equation 1 $$\Delta E=\sqrt{{\left({\Delta L}^{*}\right)}^{2}+{\left({\Delta a}^{*}\right)}^{2}+{\left({\Delta b}^{*}\right)}^{2}}$$

where ΔL*, Δa*, and Δb* are the differences in L*, a*, and b* values between the reference and test samples, respectively.

A snap test using a texture analyzer (Stable Micro Systems Ltd., UK) for a 50 kg load cell was adopted for determination of the biscuit hardness, with the application of the following settings; 5 mm/s pre-test, 3 mm/s test, and 10 mm/s post-test.

### 2.3 Nutritional quality of the formulated cricket biscuits

#### 2.3.1 Proximate analysis

The moisture, total ash, crude fiber, fat, crude protein, and carbohydrate contents of formulated biscuits were determined using standard laboratory methods (AOAC, 2010). Fat content was determined using the Soxhlet extractor (Velp SER 148, Velp Scientifica, Europe) (method 930.09). The protein content of the formulated biscuits was determined using the Kjeldahl method in a Kjeldahl analyzer (Velp UDK 159, Velp Scientifica, Europe) (method 978.04) and calculated as N x 6.25. The ash content was gravimetrically determined in a muffle furnace (Heraeus-Kundendienst, Düsseldorf, Germany) at 550 °C (method 930.05). The crude fiber was determined by acid digestion in fibre analyzer (FIWE, Velp Scientifica, Europe) and loss on ignition (method 930.10). Carbohydrate content was estimated by difference. The energy content was calculated by multiplying the protein, fat, and carbohydrate percentages by the Atwater values of 4, 9, and 4, respectively. All the analyses were carried out in triplicates.

#### 2.3.2 Mineral analysis

Mineral analysis (Ca, P, Na, Mg, Fe, Zn, Mn, Cu, and Co) of the formulated biscuits was carried out using a standard method (AOAC, 2010). Eight-milliliters of concentrated nitric acid (16.2 mol/L) (VWR Chemicals, Fontenaysous-Bois, France) and 2 mL of 9.8 mol/L hydrogen peroxide (SigmaAldrich, USA) were used to digest 0.5 g of each sample overnight in a fume chamber. This was followed by digestion at the following temperatures: 75 °C for 30 min, 120 °C for 20 min, 180 °C for 20 min, and 200 °C for 10 min in a temperature-controlled block digester (Model TE007-A, TECNAL, São Paulo, SP, Brazil). After cooling, the wet ashes were dissolved in 0.4 mol/L nitric acid and then diluted ten-fold to appropriate concentrations based on the mineral element and the resulting calibration curve. Calibration curves for quantification were obtained by also measuring standard solutions from certified stock solutions (Chem Lab, Zedelgem, Belgium) serially diluted to give calibration standards of 400, 800, 2000, and 4000 g/L. The contents of each investigated element and the standard were determined by inductively coupled plasma optical emission spectrometry (ICP-OES) measurements (Optima 4300™ DV ICP-OES, Perkin Elmer, Wellesley MA, USA). Perkin Elmer Winlab 32 software (Perkin Elmer, USA) was used for external standard calibration and data acquisition. All samples were analyzed in duplicates.

#### 2.3.3 Fatty acid analysis

Fatty acids determination followed the procedures described by Ochieng et al. (2022). Folch's oil extraction technique, using a mixture of 20 mL dichloromethane and methanol (2:1 v/v) was adopted to extract lipids from 10 g of ground biscuits. Extracted lipids of approximately 100 mg were each esterified by adding 1 mL of sodium methoxide solution (15 mg/mL) followed by vortexing for 1 min, sonicating for 10 min and incubating in a 70 °C water bath for 1 h. Distilled deionized water (100 μL) was added to quench the reaction then vortexed for another 1 min. One-milliliter of gas chromatography (GC)-grade hexane (Sigma–Aldrich, St. Louis, MO, USA) was added to extract the resulting fatty acids methyl esters (FAMEs) and centrifuged at 14,000 rpm for 20 min. The supernatant was carefully dried over anhydrous sodium sulphate and filtered. The dry supernatant (1.0 μL) was analyzed by GC–MS on a 7890A gas chromatograph linked to a 5975C mass selective detector (Agilent Technologies Inc., Santa Clara, CA, USA). The GC was equipped with a (5%-phenyl)-methylpolysiloxane (HP5 MS) low bleed capillary column (30 m × 0.25 mm i.d., 0.25 μm; J&W, Folsom, CA, USA). Helium at a flow rate of 1.25 mL/min was the carrier gas. At a rising rate of 10 °C/min, the oven temperature was programmed from 35 °C to 285 °C, with both the initial and final temperatures maintained at 5 min and 20.4 min, respectively. Both the ion source and quadrupole mass selective detector temperatures were maintained at 230 °C and 180 °C, respectively. The spectrum masses from electron impact (EI) were acquired at an acceleration energy of 70 eV and, the fragment ions analyzed over 40–550 m/z mass range in the full scan mode and the filament delay time set at 3.3 min. Octadecanoic acid (≥95% purity) (Sigma-Aldrich, St. Louis, MO) was used to prepare serial dilutions of authentic standard methyl octadecanoate (0.2–125 ng/μL) which were also analyzed by GC–MS in full scan mode to generate a linear calibration curve (peak area vs. concentration) with the following equation: $Y=\left[5\times 10^{7}x\right]+\left[2\times 10^{7}\right]$; $R^{2}=0.9997$ and used for external quantification of the various fatty acids. ChemStation B.02.02 software was used for the data acquisition and the compounds identified by comparison of mass spectral data and retention times with those of authentic standards and reference spectra published by library–MS databases: National Institute of Standards and Technology (NIST) 05, 08, and 11. Determination of the FAMEs was done in triplicates.

#### 2.3.4 Consumer acceptability

This study was conducted in Keiyo South sub-county, Elgeyo Marakwet County, Kenya. A preliminary sensory test, recruiting children and caregivers from the same demographic area but from a different locale (n = 86), suggested higher consumer preferability of biscuits formulated with 20% cricket flour (CBB20) and the control, justifying their use in the subsequent test. The study targeted 214 children aged between 5 and 10 years and 146 caregivers, tallying to 360 participants. The demographic characteristics of the adult participants were as follows: Gender distribution was 83% female and 17% male. The average monthly income was Kenya shillings (KES) 10,000. Adults aged 18 to 34 constituted the majority of the population, accounting for 84% of all adults, and the average household size was between 5 and 10 people. The majority had completed primary school (48.3%), secondary school (35.2%), and college (16%).

Before the assessment, the contents of the biscuit samples were disclosed to all the participants verbally and in writing for consent. Further, participants with history of self-reported allergy to any ingredient in the biscuits, as well as any medical contraindication, were deemed ineligible. The participants were each randomly served with two coded biscuits samples and clean water for mouth cleansing in between the tests with children participating first followed by the caregivers. The panelists were instructed to rate the color, flavor, taste, texture, and overall acceptability of the biscuits against a 5-point hedonic scale (1 = dislike very much, 5 = like very much). As a reward for participating in the experiment, participants were asked to keep the remaining biscuits. This was done to reduce the risk of in-kind endowment effects, which could have distorted the actual liking (Lagerkvist, Okello, & Karanja, 2015). Ethical approval for the involvement of human subjects in this study was granted by Jomo Kenyatta University of Agriculture and Technology Research Ethics Committee, Reference number: JKU/2/4/896B, dated 2/4/2020.

#### 2.3.5 Caregivers and children willingness to pay for cricket-based biscuits

A double-bounded dichotomous choice questionnaire, applying the contingent valuation method, was used to determine the consumer willingness to pay (WTP). The caregivers and children were recruited to provide a yes or no response based on their WTP. Depending on the responses given, follow-up questions were posed i.e., ‘yes’ respondents were offered a higher price while the ‘no’ respondents were given a lower price. Participants were also assigned different starting bids at random to reduce starting point bias. The forms were collected, data compiled and analyzed. A Stata double-bounded command was utilized to create the econometric WTP estimate using a linear function (Alejandro, 2012) as shown below;

(1a) $${WTP}_{i\left(zi,ui\right)=zi\beta +ui}$$

(2) $$u_{i∼N\left(0,\sigma^{2}\right)}$$

Where, zi = vector of explanatory variables, ui = is an error term, β= vector of parameters and constant variance, $\sigma^{2}$.

In the double-bounded model, the respondents provided two responses to the closed willingness to pay (WTP) questions, with the first bid amount being marked as *t1* and the second bid amount being marked as *t2*. The WTP was then reduced to one of the four following categories of each respondent:

$$Yes,yes\;answer=t^{2}>t^{1}and\;t^{2}\leq WTP<\infty$$

$${Yes,No\;answer=t}^{1}\leq WTP\;<t^{2}$$

$${No,yes\;answer=t}^{2}<t^{1}and\;t^{2}\leq WTP<t^{1}$$

No,noanswer=0≤WTP<tˆ2

In the model for estimating WTP, the individual *i* replies were coded with the dichotomous variables y^1^ 1 (response to the first WTP question) and y^2^ 1 (response to the second WTP question), respectively, indicating that the variables were given the value 1 if the individual's answer was yes and 0 if no. Because of the normal distribution of the WTP and error term *ui,* the probability of the four cases is defined as follows (equations (3), (4), (5), (6))),

Double-bounded model the probability that an individual answers ‘Yes’ to the first question and ‘No’ to the second can be expressed as *Pr (*$y_{i}^{1}$ *=1,* $y_{i}^{2}$ *= 0|zi) = Pr (s, n)* Where $y_{i}^{1}\text{,}$ is the response to the first question and $y_{i}^{2}\text{,}$ is the response to the second question (so, the right side of the statement omits the fact that the probability depends on the values of the explanatory factors).

When WTP*i* (*zi,ui*) = *z0iβ + ui* and *ui* ∼ N (0, σ2), Therefore, the probability of each one of the four cases is given by:

(3) $$y_{i}^{1}=1\;and\;y_{i}^{2}=1$$

$$\mathrm{pr}\left(y_{i}^{1}=1,y_{i}^{2}=1/z_{i}\right)=\mathrm{pr}\left(Y,Y\right)=\emptyset /z_{i}\left(\frac{\beta }{\sigma }-\frac{t^{2}}{\sigma }\right)\text{,}$$

(4) $$y_{i}^{1}=1\;and\;y_{i}^{2}=0$$

$$\mathrm{pr}\left(y_{i}^{1}=0,y_{i}^{2}=1/z_{i}\right)=\mathrm{pr}\left(Y,N\right)=\emptyset /z_{i}^{,}\left(\frac{\beta }{\sigma }-\frac{t^{1}}{\sigma }\right)-\emptyset \left(z_{i}^{,}\frac{\beta }{\sigma }-\frac{t^{2}}{\sigma }\right)$$

(5) $$y_{i}^{1}=0\;and\;y_{i}^{2}=1$$

$$\mathrm{pr}\left(y_{i}^{1}=0,y_{i}^{2}=1/z_{i}\right)=\mathrm{pr}\left(N,Y\right)=\emptyset /z_{i}^{,}\left(\frac{\beta }{\sigma }-\frac{t^{2}}{\sigma }\right)-\emptyset \left(z_{i}^{,}\frac{\beta }{\sigma }-\frac{t^{1}}{\sigma }\right)$$

(6) $$y_{i}^{1}=0\;and\;y_{i}^{2}=0$$

$$\mathrm{pr}\left(y_{i}^{1}=0,y_{i}^{2}=1/z_{i}\right)=\mathrm{pr}\left(N,N\right)=1-\emptyset /z_{i}^{,}\left(\frac{\beta }{\sigma }-\frac{t^{2}}{\sigma }\right)$$

The maximum likelihood method was used to estimate β and μ. The function that needs to be maximized in order to determine the model's parameter is described using equation (7).

(7) $$\sum_{i=1}^{n}=\left[d_{i}^{Y,Y}In\left(\emptyset z_{i}^{,}\frac{\beta }{\sigma }-\frac{t^{2}}{\sigma }\right)+d_{i}^{Y,N}Id_{i}^{Y,N}In\emptyset \left(z_{i}^{,}\frac{\beta }{\sigma }-/\sigma -\emptyset z_{i}^{,}\frac{\beta }{\sigma }-\frac{t^{2}}{\sigma }\right)+d_{i}^{N,Y}In\left(\emptyset \left(z_{i}^{,}\frac{\beta }{\sigma }-\frac{t^{2}}{\sigma }\right)-\emptyset z_{i}^{,}\frac{\beta }{\sigma }-\frac{t^{2}}{\sigma }\right)-\emptyset z_{i}^{,}\frac{\beta }{\sigma }-\frac{t^{2}}{\sigma }\right]$$

Where *i* = 1,. …., n, whereas $d_{i}^{Y,Y},d_{i}^{Y,N}$ N, and $d_{i}^{N,Y}$ $d_{i}^{N,N}$ N, are considered to have an indicator value of 1 or 0 for each responder in each case. As a result, we may calculate an approximate WTP after directly obtaining β, and σ.

### 2.4 Statistical analysis

All data were analyzed using R Studio version 2022.12.0 + 353.pro3 (R Core Team, 2022). One-way analysis of variance (ANOVA) was performed to establish the variabilities in the physical properties and nutritional contents of the biscuits formulated with different cricket levels. Fisher's Least Significant Difference (LSD) test was adopted for mean separation at 5% significant level. An independent student t-test was used to compare and discern the differences in consumer preference between control and 20% cricket flour-biscuits. The average willingness to pay and willingness to pay predictors were analyzed according to an interval data logit model.

## 3 Results

### 3.1 Physical properties of the formulated biscuits

The physical characteristics of the cricket-based cookies and the control biscuits are indicated in Table 2. The diameter of the biscuits ranged from 55.4 to 58.8 mm with the biscuits containing 20% cricket flour and the control exhibiting the highest and lowest diameters, respectively. Biscuits formulated with 20% cricket flour showed significantly higher spread ratio, with the control biscuits expressing the lowest. The highest thickness was observed in the control, while 20% cricket flour supplementation indicated the lowest. The hardness and fracturability of the biscuits were reduced with the incremental level of cricket flour supplementation of the biscuits.

**Table 2:** Physical properties of the formulated biscuits.

Parameters	WBB	CBB5	CBB10	CBB20	*F*_(3,36*)*_	*P-value*
Diameter (mm)	55.4 ± 0.2^c^	56.3 ± 0.4^b^	57.9 ± 0.6^b^	58.8 ± 0.5^a^	4.44	0.001
Thickness (mm)	7.2 ± 0.6^a^	6.5 ± 0.7^b^	6.5 ± 0.4^b^	6.3 ± 0.5^c^	19.34	0.01
Weight (g)	8.2 ± 0.5^a^	8.5 ± 0.7^a^	8.5 ± 0.7^a^	8.4 ± 0.7^a^	0.41	ns
Spread ratio	7.6 ± 0.2^d^	8.6 ± 0.7^c^	8.9 ± 0.5^b^	9.3 ± 0.4^a^	6.74)	0.001
Hardness (N)	46.1 ± 1.8^a^	44.8 ± 1.8^b^	44.6 ± 0.6^b^	35.0 ± 2.2^c^	41.14	0.001
Fracturability (mm)	6.3 ± 0.2^a^	6.2 ± 0.3^a^	5.8 ± 0.2^b^	5.3 ± 0.4^c^	12.74	0.001

Values are presented as Mean ± SD (*n =* 3). Means with the same superscript letters within the same row do not differ significantly (*P* > 0.05). ns = not significant. WBB- control, CBB5, CBB10, and CBB20 represent biscuits supplemented with 0%, 5%, 10% and 20% of cricket flour, respectively.

### 3.2 Color attribute of the formulated biscuits

**Fig. 1:**
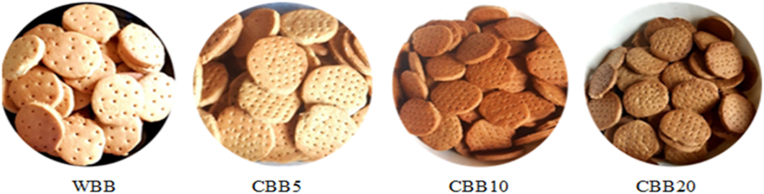
The physical appearance of the developed biscuits. WBB (Control or reference biscuits); CBB5, CBB10, and CBB20 (Biscuits enriched with 5, 10, and 20% of cricket flour, respectively.

Present results display significantly increase in redness (a*) of the biscuits while reducing their lightness (L*) and yellowness (b*) color parameters with increasing cricket flour inclusion (Table 3 and Fig. 1).

**Table 3:** Color parameters (L*, a*, b*, Chroma and ΔE) of control biscuits (0% cricket powder inclusion) and biscuits enriched with 5%, 10% and 20% cricket flour.

Parameter	WBB	CBB5	CBB10	CBB20	*F*_*(3,16)*_	*P-value*
L*	74.55 ± 0.22^a^	69.66 ± 0.20^b^	62.21 ± 0.23^c^	53.45 ± 0.09^d^	159.40	0.001
a*	6.22 ± 0.05^d^	6.77 ± 0.08^c^	6.92 ± 0.42^b^	7.22 ± 0.33^a^	19.80	0.001
b*	24.88 ± 0.36^a^	22.73 ± 0.36^b^	20.88 ± 0.12^c^	19.99 ± 0.16^d^	882.92	0.001
***ΔE***	–	5.11 ± 0.00^c^	12.99 ± 0.41^b^	21.66 ± 0.01^a^	97.35	0.001

Values are presented as Mean ± SD (*n =* 3). Different superscripts within the same row indicate a significant difference (*P* < 0.05). L*- ranging from 0 (black) to 100 (white) chromaticity; a*-Green-red chromaticity; b*- blue-yellow chromaticity; WBB- control, CBB5, CBB10, and CBB20 represent biscuits supplemented with 0%, 5%, 10 and 20% of cricket flour, respectively.

### 3.3 Nutritional quality of cricket-based biscuits

#### 3.3.1 Proximate composition and energy value

The proximate composition and energy values exhibited significant variabilities with increasing cricket flour incorporation (Table 4). The levels of protein, ash, fiber, fats, and energy expressed an increasing trend correlating with cricket flour addition.

**Table 4:** Proximate composition (g/100 g) and energy levels (Kcal) of formulated biscuits.

Component	WBB	CBB5	CBB10	CBB20	*F*_(3,8)_	*P-value*
Protein	9.55 ± 0.08^d^	11.84 ± 0.21^c^	13.11 ± 0.08^b^	16.12 ± 0.21^a^	45.04	0.001
Ash	0.34 ± 0.03^b^	1.00 ± 0.07^b^	1.11 ± 0.31^a^	1.12 ± 0.09^a^	7.60	0.01
Fiber	0.64 + 0.02^d^	1.12 ± 0.02^b^	1.53 + 0.02^c^	1.81 ± 0.02^a^	46.92	0.001
Carbohydrate	68.99 ± 2.45^a^	63.99 ± 3.12^c^	61.44 ± 2.45^c^	56.55 ± 3.12^d^	15.83	0.001
Fats	18.60 ± 0.40^c^	20.66 ± 0.10^b^	20.93 ± 0.31^b^	22.44 ± 0.40^a^	15.83	0.001
Moisture	1.88 ± 0.49^a^	1.77 ± 0.21^a^	2.11 ± 0.31^a^	1.88 ± 0.19^a^	0.87	ns
Energy	480.88 ± 13.21^c^	487.88 ± 20.43^b^	486.01 ± 9.42^b^	492.55 ± 12.80^a^	0.65	0.01

Values are presented as Mean ± SD (*n =* 3). Different superscripts within the same row indicate a significant difference (*P* < 0.05). WBB, CBB5, CBB10, and CBB20 represent biscuits fortified with 0, 5%, 10 and 20% of cricket flour, respectively.

#### 3.3.2 Mineral composition

Substantial significant differences were discerned in the concentrations of all the minerals with increasing cricket flour supplementation (Table 5). Supplementing the biscuits with rising levels of cricket flour proportionally adjusted the concentrations of all the minerals with 20% cricket biscuits expressing the highest concentrations.

**Table 5:** Mineral composition (mg/100g DM) of biscuits formulated with cricket flour.

Minerals	WBB	CBB5	CBB10	CBB20	*F*_*(3,8)*_	*P-value*
Ca	39.80 ± 7.82^d^	114.10 ± 6.5^c^	137.11 ± 8.98^b^	149.10 ± 10.39^a^	23.01	0.001
P	342.61 ± 24.47^c^	465.60 ± 21.50^b^	516.63 ± 23.30^a^	515.94 ± 15.46^a^	6.39	0.001
K	673.50 ± 27.98^b^	761.60 ± 20.32^ab^	1200.03 ± 17.09^a^	1249.04 ± 20.27^a^	16.32	0.001
Mg	118.24 ± 8.17	135.33 ± 7.39^c^	211.83 ± 11.99^b^	227.66 ± 17.72^a^	142.46	0.001
Na	99.77 ± 10.33^c^	103.51 ± 10.29^b^	116.51 ± 10.02^a^	119.24 ± 8.72^a^	12.29	0.01
Fe	1.33 ± 0.07^d^	1.40 ± 0.02^c^	1.42 ± 0.04^b^	1.55 ± 0.08^a^	0.48	0.01
Mn	13.22 ± 0.91^a^	13.63 ± 1.19^a^	14.00 ± 0.75^a^	14.50 ± 0 .79^a^	0.07	ns
Zn	4.23 ± 0.28^d^	7.22 ± 0.75^c^	11.66 ± 4.07 ^b^	16.88 ± 2.39^a^	59.75	0.001
Cu	2.01 ± 0.16^d^	2.94 ± 0.08^c^	7.63 ± 0.55^b^	9.53 ± 0.61^a^	20.96	0.001
Co	0.04 ± 0.00^a^	0.05 ± 0.00^a^	0.05 ± 0.00^a^	0.31 ± 0.00^a^	0.80	0.001

In the same row, mean ± SD (*n =* 3) followed by different lowercase superscript letters are significantly different at *P* ≤ 0.05. WBB, CBB5, CBB10, and CBB20 represent biscuits fortified with 0, 5%, 10 and 20% of cricket flour, respectively.

#### 3.3.3 Fatty acids profile

The fatty acid composition of the biscuits was significantly (*p* < 0.05) altered as a result of the use of cricket flour in the recipe (Table 6). A total of 21 fatty acids were detected, with saturated (SFA), monounsaturated (MUFA) and polyunsaturated fatty acids (PUFA) accounting for 35.83–50.41%, 47.09–58.92% and 2.51–6.86%, respectively across the formulated biscuits. Methyl hexadecanoate and methyl octadecanoate of the SFAs, methyl (9*Z*)-octadecenoate and methyl (11Z)-octadecenoate of the MUFAs and methyl (9Z, 12Z)-octadecadienoate of the PUFAs were the predominant fatty acids (FAs) detected in the biscuit samples.

**Table 6:** Fatty acid composition (μg/mg) of oil extracted from the formulated biscuits.

FAMEs	Corresponding Fatty Acid	ω-n (Δn)	WBB	CBB5	CBB10	CBB20	*F*_*(3,8)*_	*P-value*
Methyl undecanoate	Undecyclic acid	**C11:0**	N.D.	N.D.	N.D.	0.01 ± 0.02	- N.D.	N.D.
Methyl dodecanoate	Lauric acid	**C12:0**	0.55 ± 0.20^d^	0.94 ± 0.76^c^	1.04 ± 0.51^b^	1.93 ± 0.27^a^	35.24	0.01
Methyl tetradecanoate	Myristic acid	**C14:0**	0.84 ± 0.02^b^	0.99 ± 0.52^b^	0.92 ± 0.43^b^	1.55 ± 0.25^a^	17.49	0.01
Methyl hexadecanoate	Palmitic acid	**C16:0)**	34.84 ± 0.78^a^	13.44 ± 0.42^b^	1.00 ± 0.09^d^	5.22 ± 0.67^c^	823.32	0.001
Methyl octadecanoate	Stearic acid	**C18:0**	3.65 ± 0.47^d^	21.22 ± 0.04^b^	28.24 ± 0.43^a^	19.56 ± 0.84^c^	66.81	0.001
Methyl nonadecanoate	Nonadecyclic acid	**C19:0**	0.10 ± 0.28^c^	1.11 ± 0.05^a^	0.22 ± 1.36^b^	0.21 ± 0.03^b^	23.59	0.001
Methyl eicosanoate	Arachidic acid	**C20:0**	1.67 ± 0.59^b^	2.22 ± 0.60^a^	1.77 ± 0.51^b^	0.88 ± 2.71^c^	24.90	0.001
Methyl heneicosanoate	Heneicosylic acid	**C21:0**	N.D.	0.22 ± 0.68^c^	1.11 ± 0.39^b^	1.74 ± 0.32^a^	40.29	0.01
Methyl docosanoate	Behenic acid	**C22:0**	3.32 ± 0.42^a^	1.42 ± 0.34^c^	0.81 ± 0.82^d^	1.11 ± 0.57^b^	60.91	0.01
Methyl tricosanoate	Tricosylic acid	**C23:0**	N.D.	2.01 ± 0.78^a^	0.30 ± 0.04^c^	1.11 ± 0.02^b^	28.58	0.001
Methyl tetracosanoate	Lignoceric acid	**C24:0**	N.D.	0.66 ± 0.25^c^	0.63 ± 0.11^b^	1.51 ± 0.08^a^	13.46	0.001
		**Σ SFA**	44.97^a^	43.23^b^	36.04^c^	34.83^d^		
Methyl (9Z)-hexadecanoate	Palmitoleic acid	**C16:1(n-7)**	0.44 ± 0.66^d^	6.88 ± 0.38^a^	2.02 ± 0.63^c^	3.52 ± 0.84^b^	173.69	0.001
Methyl 10Z-heptadecenoate	(10*Z*)-Heptadecenoic acid	**C17:1(n-7)**	0.32 ± 0.30^c^	1.04 ± 0.16^a^	0.33 ± 0.24^d^	0.89 ± 0.66^b^	9.39	0.01
Methyl (9*Z*)-Octadecenoate	Oleic acid	**C18:1(n-9)**	40.94 ± 0.82^a^	4.23 ± 0.11^d^	4.60 ± 0.37^b^	4.42 ± 0.89^c^	610.29	0.001
Methyl (11Z)-octadecenoate	Vaccenic acid	**C18:1(n-11)**	3.80 ± 0.29^c^	32.98 ± 0.28^b^	47.56 ± 0.69^a^	49.89 ± 0.88^a^	584.80	0.001
Methyl 9Z-eicosenoate	Gondoic acid	**C20:1(n-9)**	1.77 ± 0.49^c^	3.33 ± 0.80^b^	4.03 ± 0.89^a^	0.66 ± 0.65^d^	155.72	0.001
		**Σ MUFA**	47.27^d^	48.46^c^	58.54^b^	59.38^a^		
Methyl (9Z,12Z)- octadecadienoate	Linoleic acid	**C18:2(n-6)**	2.51 ± 0.68^b^	6.06 ± 0.08^a^	3.23 ± 0.53^b^	3.44 ± 0.62^b^	14.92	0.001
Methyl (9Z,12Z,15Z)-octadecatrienoate	α-Linoleic acid	**C18:3(n-3)**	N.D.	N.D.	3.24 ± 0.99^a^	3.10 ± 0.23^b^	488.67	0.001
		**Σ PUFA**	2.51^d^	6.06^c^	6.47^b^	6.54^a^		
		**PUFA/SFA**	0.06	0.14	0.18	0.19		
		**n-6/n-3**	N.D.	N.D.	1.0	1.1		

SFA Saturated Fatty Acids; MUFA – Monounsaturated Fatty Acids; PUFA – Polyunsaturated Fatty Acids. In the same row, means ± SD (*n =* 3) followed by different lowercase letters are significantly different at *P* ≤ 0.05. Control, CBB5, CBB10, and CBB20 represent biscuits fortified with 0, 5%, 10 and 20% of cricket flour, respectively. N.D. = not determined.

### 3.4 Consumer acceptability and willingness to pay for cricket-based biscuits

#### 3.4.1 Consumer acceptability of CBB20 and WBB

The sensory results indicated that all the attributes except for taste, showed significant differences between the two biscuit groups (Table 7). Color and texture of the CBB20 were ranked lower than the control cookies (WBB) served to the children contrariwise to the caregivers’ rankings.

**Table 7:** Independent *t*-test of significance of differences in score of WBB and CBB20.

Children					Caregivers		
Attribute	Biscuits	Mean ± SD	t-value (*df* = 214)	*p*-value	Mean ± SD	t-value (*df* = 146)	*p*-value
Color	CBB20	3.57 ± 1.43	−6.65	0.000	4.52 ± 1.53	7.65	0.000
	WBB	4.43 ± 0.73			3.57 ± 0.79		
Aroma	CBB20	3.67 ± 1.13	4.13	0.000	3.43 ± 1.46	−4.53	0.000
	WBB	3.14 ± 1.25			4.14 ± 1.47		
Taste	CBB20	3.95 ± 1.21	−0.71	0.478	4.12 ± 0.39	−1.68	0.970
	WBB	4.05 ± 1.21			4.19 ± 0.27		
Texture	CBB20	3.62 ± 1.76	−4.35	0.000	4.71 ± 1.31	10.07	0.000
	WBB	4.33 ± 1.21			3.71 ± 1.43		
Overall acceptance	CBB20	4.86 ± 0.35	8.11	0.000	3.86 ± 0.43	−8.00	0.000
	WBB	4.33 ± 0.84			4.76 ± 1.32		

WBB-Control biscuits; CBB20-Biscuits formulated with 20% cricket flour.

#### 3.4.2 Caregivers’ willingness to pay for cricket-based biscuits

The analysis of logical progression of questions employed in a double-bounded dichotomous choice questionnaire to generate consumers’ willingness to pay when offered different bids are depicted in Table 8. About 67.4% of the respondents agreed to pay the two given bids of Kenya shillings (KES) 20–30 whereas 26.3% of respondents declined paying the highest group amount of KES 50–60 (USD 0.40–0.49).

**Table 8:** Caregivers’ responses to the two suggested bids (DC).

1st Bid	2nd Bid higher	3rd Bid lower	Yes/Yes	Yes/No	Yes/No	No/No
30	40	20	67.39%	10.86%	13.04%	8.69%
40	50	30	37.93%	17.24%	31.03%	13.79%
50	60	40	24.24%	21.21%	30.30%	24%
60	70	50	13.15%	23.68%	36.86%	26.31%

#### 3.4.3 Factors hindering appreciation for cricket-based biscuits

A larger share of the respondents (30.8%) was unwilling to consume the biscuits, citing “it is not usual to consume insects in my society,” for the rejection. Approximately 23.1% of the respondents rejected the cricket biscuits because of lack of interest in the insect proteins and aversion. A group, accounting for 15.4% of the total respondents avoided the cricket biscuits due to financial constraints. This group may have liked the cricket biscuits but were unable to afford them. Another group comprising 7.6% of the respondents were skeptical of the new food. All these reasons for negative WTP could be a precedent for future actions, such as educating consumers about the benefits of edible insects.

#### 3.4.4 Factors affecting the willingness to pay for cricket-based biscuits

The respondents' WTP was a significantly influenced by age, income, education, gender, and employment (Table 9). There also existed significant negative correlation between the bid amount and the respondents' willingness to pay.

**Table 9:** Factors affecting the Willingness to pay for cricket-based biscuits.

Attribute	Coefficient	SE	P-value
Bid Amount	−0.02***	0.03	0.000
Gender	13.64***	6.89	0.009
Age	5.02**	2.55	0.017
Education Level 1	−0.30	−0.05	0.340
Education Level 2	0.05	−0.01	0.871
Education Level 3	0.41	−0.08	0.271
Income Level KES 15,000–10,000	−0.58**	0.11	0.017
Income Level KES 210,000–15,000	−0.56**	0.11	0.019
Income Level KES 315,000–20,000	−1.77***	0.31	0.000
Marital status	1.92	5.56	0.729
Employment	−0.68**	0.05	0.012
Household number	−5041808	3.70	0.892
Constant	9.94	24.92	0.069
n		117	
Log likelihood		−148.67	

#### 3.4.5 Estimated willingness to pay for cricket-based biscuits

To get the mean WTP (Table 10), the coefficient of the restricted equation was calculated as an initial step without consumer characteristics. An estimated mean WTP was then computed to give KES 37 (USD 0.34) per 100 g of biscuits.

**Table 10:** Mean WTP for cricket-based biscuits.

Variables	Estimate	Standard error	P-value
Constant(α)	5.03	0.34	<0.001
Bids(ρ)	0.14	0.06	<0.001
Mean WTP (α/ρ)	36.82		
Number of observations	146		
Log-likelihood	481451		
Chi-squared	3532		

## 4 Discussions

The higher diameter of cricket-based biscuits could be attributed to the low gluten content caused by the dilution effect of cricket flour in the dough, which could have altered the consistency and structure of the dough (Mudgil, Barak, & Khatkar, 2017), resulting in meagerly deformed biscuits. Conversely, higher dough gluten levels have been found to distort the shape of end products during preparation and baking (Smarzyński et al., 2021). Likewise, cricket flour inclusion levels also correlated with the biscuit spread ratio. The presence of the cricket flour in the dough mix disoriented the gluten protein-carbohydrates linkages by weakening the bond networks, producing dough with lower compactness (Smarzyński et al., 2021). Similar findings have been made by other researchers (Akande, Falade, Badejo, & Adekoya, 2020). Spread ratio is a quality parameter in biscuits that positively correlates with their desirability (Klunklin & Savage, 2018).

While there was no significant difference in weight, the developed biscuits' thickness varied significantly between formulations. The progressive increase in thickness with inclusion levels may be attributed to the addition of cricket flour which reduced gluten and starch levels. The alteration of such components of wheat has a substantial impact on dough rheology and product quality. Compared to wheat dough, low gluten dough has a reduced cohesion and elasticity (Cappelli, Oliva, Bonaccorsi, Lorini, & Cini, 2020). These parameters are crucial during the production of biscuits because dimensional variables impact on both the textural characteristics and the package development and design process (Irungu et al., 2018).

The increasing cricket flour levels also reduced the hardness and fracturability of the biscuits rendering them softer and less brittle. Incorporation of cricket flour into the biscuit dough introduced novel proteins which suppressed the levels of gluten and starch (Pauter et al., 2018), resulting in weaker and less elastic dough (Sinthusamran, Benjakul, Kijroongrojana, & Prodpran, 2019), reducing the biscuits' hardness and fracturability. Further, since the amount of amylopectin and amylose in baked goods is associated with their hardness, as novel protein concentrations increased, the starch levels fell, thereby contributing to the reduction in the hardness of the biscuits (Biró et al., 2020). Therefore, the greater hardness of the control biscuits is explicable by the unaltered strength of the gluten network and undiluted starch levels (Pauter et al., 2018). Similar results were found by Smarzyński et al. (2021), who found that replacing wheat flour with other flours resulted in a reduction in the hardness of the biscuits. In contrast Bawa, Songsermpong, Kaewtapee, and Chanput (2020), who used eggs and unrefined wheat flour as ingredients in their study, observed that increasing the amount of cricket flour in cookie dough enhanced the biscuits' hardness.

Sensory properties like color influence the alimentary behavior of consumers, determining their choice of food. The cricket powder levels in the biscuits influenced the color parameters of the resultant biscuits by enhancing the redness (a*) but reducing their lightness (L*) and yellowness (b*). Bawa et al. (2020) also deduced concurring findings on cricket-enriched bakery products. Likewise, significant differences were also realized in the total color difference (***Δ***E) of the cricket-cookies with 20% cricket biscuits exhibiting the highest value. These differences portray color changes from slightly dark color to darker colors with increasing cricket inclusion. This can be attributed to the possible addition of highly reactive free amino acids and proteins introduced from cricket flour, which may have caused intense Maillard reactions, generating dark colored biscuits after baking (Smarzyński et al., 2021).

The progressive increase in the proportions of protein, ash, fiber, fats, and energy of the biscuits with cricket substitution levels may be linked to their additive supplies from cricket flour, previously reported to be endowed with appreciable amounts of these proximate parameters (Magara et al., 2021). In particular, the increase in protein content from the control to the 20% cricket-biscuits accounted for 70%, revealing the possible contribution of cricket flour reportedly containing 55–73% (Magara et al., 2021), in the production of nutritious biscuits. Similar trend was manifested when other researchers supplemented baked products with insect flour (Zielińska, Pankiewicz, & Sujka, 2021). This strategy is nutritionally crucial in combating protein deficiency in developing nations such as Kenya (World Health Organization and Food and Agriculture Organization, 2018), by developing such nutrient dense insect-based products compared to their conventional counterparts formulated chiefly with nutritionally inferior ingredients like wheat. Protein is an essential nutrient for the proper growth and development of children (Agbemafle, 2020). The reducing trend in carbohydrate levels from the control to the 20% cricket-biscuits is attributable to the enrichment with the low carbohydrate cricket flour (Bawa et al., 2020). In as much as carbohydrates contribute to the total calories when computed, the energy levels steadily rose with the cricket flour addition, suggesting the possible influence of the fat content of cricket flour.

The minerals phosphorous, calcium, potassium, magnesium, and sodium were the most predominant minerals, which have also been previously reported in different cricket species (Magara et al., 2021). Evidently, the enhanced mineral levels may be ascribed to formulation of the biscuits with mineral rich cricket flour, as has been demonstrated in another study (Smarzyński et al., 2021). The developed cricket-based biscuits can therefore contribute significantly to the recommended dietary intakes (RDA) of most of the nutritionally essential minerals. For instance, the biscuits may contribute 10–10.5%, 47.8–111.9%, 11.4–14.9 and 66.5–73.7% RDIs of iron, zinc, calcium and phosphorous, respectively as set by FAO/WHO. Since mineral deficiencies, especially calcium, iron, and zinc, are still a major concern in developing countries (Harika et al., 2017) leading to rampant cases of illnesses such as osteoporosis and anemia (Cormick & Belizan, 2019), their intakes in early life may serve as a preventive measure. Therefore, food products similar to cricket biscuits developed in this study can be key to alleviating micronutrient deficiencies among children and women.

The dominant SFAs, MUFAs and PUFAs detected in this study are consistent with studies which identified palmitic acid, stearic acid, oleic acid and linoleic acids as the abundant fatty acids in cricket flour, considerably enhancing their respective levels in the bakery products they are used to formulate (Kowalczewski et al., 2021). Certain SFAs such as methyl dodecanoate, methly tetradecanoate, methly octadecanoate, methyl heneicosanoate and methly tetracosanoate also indicated a similar trend. It is therefore admissible to conclude that, the haphazard variabilities in the proportions of the different fatty acids are dependable on their shares in the formulation levels of cricket flours. Surprisingly, the total PUFAs and MUFAs demonstrated an increasing tendency correlating with insect inclusion levels. This observation can be underpinned by a previous report which cited the richness of cricket flours in unsaturated fatty acids (Ghosh, Gao, Thakur, Siu, & Lai, 2017). Therefore, adding cricket powder to the biscuits progressively enhanced their levels. Methyl (9Z, 12Z, 15Z)-octadecatrienoate was the only omega 3 detected only in CBB10 and CBB20 biscuits. Its non-detection in the control biscuits connotes that their source was only from cricket powder ingredients. This further underscores the significant contribution of edible insects as ingredients with the potential of ameliorating human health through the production of cardio-friendly products. In as much as these fats serve as nutrients sources in the baked products, they are also derivatized into hydroperoxides during baking yielding aroma compounds (Kowalczewski et al., 2021) that ultimately influence their sensory acceptance.

The low rating of color among the children can be attributed to an unexpectedly dark color change, occasioned by Maillard reaction between dough sugars and free amino acids from cricket flour, which did not match the snack they are accustomed to. The higher desirability of the color and texture of CBB20 among the caregivers is a demonstration that the dark color and gritty texture had no effect on their opinions. This could be due to the notion that brown-colored and fibrous foods are more nutritious and wholesome (Pauter et al., 2018). As established by Homann et al. (2017), color and aroma are more important attributes than nutritional contents as they are perceived before food intake and dictate food adoption (Amanda, Heary, Nixon, & Kelly, 2010). Interestingly, cricket-based biscuits were generally more acceptable than the control among the children. Similar ratings of insect based products were demonstrated when bread were supplemented with meal worm powder (Cappelli et al., 2020), sorghum biscuits formulated with termites (Cicatiello, Vitali, & Lacetera, 2020) and chocolate bars integrated with powdered insect. This implies that hiding insects into modern products is an incredible strategy of breaking the hurdles of insect aversion while promoting entomophagy (Tuccillo, Marino, & Torri, 2020).

In general, slightly more than half of consumers were willing to pay a premium price. The significant numbers of consumers prepared to pay low premiums indicate that consumers may have lacked confidence in the usefulness of cricket-based biscuits in combating malnutrition and food insecurity, as mentioned in our study information. In addition, consumers may not have trusted the nutritional information given considering the fact that protein in the biscuits is an invisible component. Our initial price of Kenya shillings (KES) 30 (USD 0.98) may also have been too high for local consumers to afford at that time of the study.

This study revealed that females would be willing to pay more for cricket-based biscuits than males. This is because women are responsible for purchasing food and cooking for the entire home and would sacrifice to give out more of their income to ensure a healthy diet for their households (Gómez-Luciano, de Aguiar, Vriesekoop, & Urbano, 2019). Conversely, earlier surveys have however reported male respondents to be more likely to consume insects than females (Giotis & Drichoutis, 2021). Age significantly and positively affected (*p* < 0.05) WFP with the middle-aged consumers (23–35 years) being more willing to pay than older consumers. Younger parents prefer quality food for their children to consume than older respondents with grown children. Similarly, readiness to adopt insects was found to be stronger among younger consumers compared to older consumers in a previous study (Verbeke, 2015). Incomes negatively correlated with willingness to pay with high income caregivers (KSH 15,000 and 20,000) not willing to pay for cricket-based biscuits. The employment coefficient exhibited an unexpected significant negative correlation with the WFP (*p*<0.005). This may be attributed to the fact that employed individuals are mostly educated and may view insect products as inferior foods meant for the poor. The computed premium price for cricket-based biscuits superseded the price of the familiar product selling at KES 30 (USD 0.27) for 100 g. Concurring results were reported by Alemu, Olsen, Vedel, Pambo, and Owino (2015) who found that consumers were willing to pay more for termite-based products with high nutritional value. This is probably attributable to the fact that consumers were informed about the nutritional benefits of edible insects before the study.

Despite the effectiveness of *G. bimaculatus* in enhancing the nutritional levels of the cricket-based biscuits as demonstrated in this study, their contribution to health through the purveyance of therapeutic compounds cannot be trivialized. Earlier reports have implicated *G. bimaculatus* extracts as effective remedies to certain medical disorders and gut health improvement (Kim, Kim, Kim, Park, & Han, 2023; Tran, Lee, & Lee, 2023). These aspects could valorize *G. bimaculatus* as a novel functional food with immense potential for alleviating malnutrition as well as advancing human health simultaneously.

## 5 Conclusion

This study has demonstrated that the substitution of wheat flour with cricket flour yielded nutritionally enriched biscuits with good sensory acceptability. The use of cricket flour to improve familiar products like biscuits could increase the diversification of insect food products, promote insect consumption, and improve household nutrition and health. However, amidst global exponential rise in adoption of insects and insect-derived products, particular attention should be paid to food frauds with regards to mislabeling of insect-enriched products. Therefore, proper control methods such as the use of near infrared spectroscopy for authentication of the insect-based products before introduction to the market should be employed to curb such vice in the food industry. Further studies on cost benefit analysis of these novel products would be crucial.

## CRediT authorship contribution statement

**Divina Arama:** Conceptualization, Methodology, Formal analysis, Investigation, Data curation, Writing – review & editing, Visualization. **John Kinyuru:** Conceptualization, Methodology, Visualization, Validation, Investigation, Writing – review & editing, Supervision. **Jeremiah Ng'ang'a:** Conceptualization, Validation, Writing – review & editing, Supervision. **Beatrice Kiage-Mokua:** Conceptualization, Methodology, Investigation, Writing – review & editing, Supervision. **Brian O. Ochieng:** Methodology, Formal analysis, Writing – review & editing. **Chrysantus Mbi Tanga:** Conceptualization, Methodology, Investigation, Validation, Resources, Writing – review & editing, Visualization, Supervision, Project administration, Funding acquisition, All authors read, reviewed, and approved the final manuscript.

## Declaration of competing interest

The authors declare that they have no known competing financial interests or personal relationships that could have appeared to influence the work reported in this paper.

## Data Availability

Data will be made available on request.
